# Dynamic network and own effects on abnormal returns: evidence from China’s stock market

**DOI:** 10.1007/s00181-020-01979-0

**Published:** 2020-12-06

**Authors:** Peter H. Egger, Jiaqing Zhu

**Affiliations:** 1 ETH Zurich, Zurich, Switzerland; 2 CEPR, London, UK; 3 CESifo, Munich, Germany; 4 Southern China Institute of Fortune Management Research, Guangdong University of Foreign Studies, Guangzhou, China

**Keywords:** Cumulative abnormal returns, Listed firms, Spillovers, Spatial and network models, Panel models, C21, C33, F15, F23, G14

## Abstract

This paper addresses the question of how to model the process of abnormal returns on individual stocks. It postulates a framework, where abnormal returns are generated by a process which features two autoregressive components, one stock-specific and one related to network effects. This process deviates from customary ones in that the parameters are specific to each stock/firm, that the autoregressive process is explicitly modelled instead of using cumulative abnormal returns over a pre-specified window, and that network effects are present. Abandoning either one of those deviations is rejected by data on Chinese stocks in 2018 and 2019, an episode which is significant for an abnormal stock-market returns analysis, as it was characterized by numerous tariff-setting events related to the “trade war” between the USA and China.

## Introduction

A recent strand of research in economics bridges the interests in financial and international economics by considering responses of stock-market prices to shocks in the announcements of trade or investment liberalizations or de-liberalizations. Examples of such work include Thompson (1993), Breinlich (2014), Moser and Rose (2014), Alfaro et al. (2017), Breinlich et al. (2018), Davies and Studnicka (2018), and Egger and Zhu (2019).

Related analyses proceed in two steps. First, a pre-event period is specified and the fundamental parameters governing the process determining the prices of individual stocks are estimated. Typically, this window spans about one trading year and is placed sufficiently many days prior to a policy event of interest to make an anticipation of policy effects unlikely. The respective process determines both *normal* (predicted) and *abnormal* (residual) stock-market returns for each stock. One key determinant of stock returns is the return on the national aggregate stock-market index. In a second step, the abnormal returns are used (as such or accumulated over a number of days, dubbed *cumulative abnormal returns*) and regressed on liberalization or deliberalization indicators of which trade–agreement membership, investment–agreement membership, tariffs, and other variables are leading examples of. The latter estimation is typically done in a relatively short time window after a policy event.

Two leading paradigms govern this type of work. First and to us most importantly, one assumption is that, conditional on average returns in a market as well as on the mentioned liberalization or deliberalization indicators, individual abnormal returns are independently distributed between the stocks in a market. Second, in case of any sluggish responses, e.g., due to adjustment costs, it is sufficient to consider cumulative abnormal returns without specifying the adjustment process.

In the present paper, we part with those assumptions by specifying abnormal returns as an autoregressive process, which features two autoregressive components: one idiosyncratic, pertaining to a stock’s own sluggish adjustment, and one related to network interdependencies between stocks. We utilize 19 protectionist-tariff-setting events associated with what is called the US–Chinese “trade war” and daily individual stock-market data for China to estimate this process. Three results stand out in comparison to earlier work: First, individual stocks tend to display a sluggish adjustment to shocks such as reflected in trade-war tariff announcements or implementations, and this sluggishness varies to a nontrivial degree between stocks/companies; second, there are important network effects among stocks which are related to input–output linkages between companies, and these network effects add to the sluggish adjustment and also vary largely across stocks/companies; finally, even the direct effects of tariffs display a relatively large variation among stocks/companies. Assuming that the parameters associated with these stock-specific responses are common to all stocks in China is rejected by the data, as is the assumption that either own dynamic or network dynamic effects are absent. These results suggest that future work focused on the explanation of abnormal returns at stock markets should pay greater attention to stock-specific aspects as well as network effects. The results in the present paper suggest that effect estimates of shocks of interest—here, increases in tariffs—may be largely biased in terms of a short-run or long-run response interpretation when disregarding the factors addressed in this paper.

The remainder of the manuscript is organized as follows. The subsequent section outlines briefly the customary approach to estimate abnormal stock-market returns, which we employ here as well. Section 3 is devoted to the measurement of inter-stock linkages through input–output relationships between companies. Section 4 outlines processes which can be used to model abnormal returns. Section 5 summarizes the data sources as well as the variables used in the analysis. Section 6 provides a condensed discussion of the main findings, and the last section concludes with a brief summary.

## Estimating abnormal returns

Let us use $i=1,\ldots ,F$ to denote firms, $e=1,\ldots ,E$ to denote event dates, and $t=1,\ldots ,T$ to denote time which are typically days. Then, changes in firms’ stock-market returns are typically modelled as a function of time-invariant, firm-specific characteristics and time-specific common characteristics which also matter in a firm specific way. Using $r_{i,e+t}$ to denote the stock-market return of *i* at day $e+t$, $m_{e+t}$ for the (common) market return, and $ex_{e+t}$ for the import-share-weighted nominal exchange rate of China, a customary model of stock-market returns is

1 $$\begin{aligned} r_{i,e+t}=\alpha _i+\zeta _{1i} m_{e+t}+\zeta _{2i} ex_{e+t}+u_{i,e+t},\quad t\in [w_1,w_2], \end{aligned}$$

where $\alpha _i$ is a stock-specific constant, $\zeta _{1i}$ and $\zeta _{2i}$ are stock-specific slope parameters, and $[w_1,w_2]$ is the estimation window for the event study. Examples of estimates of this and similar equations are found in O’Hara and Shaw (1990), Breinlich (2014), Moser and Rose (2014), Moenninghoff et al. (2015), Alfaro et al. (2017), Dewenter and Riddick (2018), or Egger and Zhu (2019).

Note that everything in Eq. (1) varies by stock/firm so that the results are quite hard to summarize and report. However, a large branch of the literature is less interested in the parameters $(\alpha _i,\zeta _{1i},\zeta _{2i})$ but in the residual, $u_{i,e+t}$, which is reflective of the *abnormal returns* of a stock or company. In what follows, we will focus on estimates of $u_{i,e+t}$ using an estimation window of $[e-610,e-361]$ (i.e., $t\in [-610,-361]$),^1^ and consider customary processes determining it so as to gauge effects of trade liberalization or deliberalization on it. Toward establishing such processes, it will be useful to devote a separate subsection to the setup of the spillover matrix and another one to the process of abnormal returns.

## Parameterizing the intensity of inter-stock spillovers

We will make use of an $F\times F$ identity matrix, $I_F$, and $H\times 1$ vectors as well as $H\times H$ matrices of ones, $\iota _H$ and $J_H$, respectively.^2^ With regard to the latter $H\in \{S,FS\}$ indicates vectors and matrices of dimension *S* and *FS*, respectively, where *S* indicates the number of sectors that the *F* firms together operate in, and *FS* is just the number of firms times the number of sectors. One useful matrix that will be used is

2 $$\begin{aligned} K_{FS}= & {} (J_{F}-I_{F})\otimes J_{S}, \end{aligned}$$

where $\otimes$ is the Kronecker product. This matrix serves the purpose of summing all elements of postmultiplied vectors except for ones pertaining to the same firm.^3^

Moreover, we will make use of an $FS\times 1$ vector of output shares, $h_{FS}$, where a typical element states how much firm *i* makes operating income with sector-*s*-type output relative to the sector’s total operating income. Based on the latter, we define the matrix

3 $$\begin{aligned} H_{FS}= & {} h_{FS}h^{\prime }_{FS}. \end{aligned}$$

After defining the Chinese sector-*S*-to-sector-*S* input–output matrix, $O_{S}$, we can define the matrix $J_{F}\otimes O_{S}$ to arrive at the weighted input–output matrix

4 $$\begin{aligned} M_{FS}= & {} H_{FS}\circ (J_{F}\otimes O_{S}) \end{aligned}$$

where $\circ$ is the Hadamard (element-wise) product, and the firm sector–firm sector input–output matrix

5 $$\begin{aligned} O_{FS}= & {} M_{FS}\circ K_{FS}. \end{aligned}$$

After defining an $FS\times F$ matrix

6 $$\begin{aligned} Z_{FS}= & {} I_{F}\otimes \iota _{S}, \end{aligned}$$

which computes sums of post-multiplied vectors across all firms for each firm and sector and assigns that sum in all rows of a firm and sector, we may define the $F\times F$ firm-to-firm input–output matrix

7 $$\begin{aligned} O_{F}= & {} Z^{\prime }_{FS}O_{FS}Z_{FS}. \end{aligned}$$

Of the latter, we will extract matrices of format $F_{e}\times F_{e}$, which we call $O_{F_e}$, where $F_{e}\le F$ is an event-*e*-specific number of firms which can at most be as large as the unique number of firms in the data across all events. $O_{F_e}$ captures the weighted impact of all firms present around event *e*, which is the same for all *t* days pertaining to that event. We will refer to the counterpart to $O_{F_e}$ which has normalized entries so that the elements in each row sum up to unity by $W_{F_e}$, which is also of format $F_{e}\times F_{e}$.^4^

## The process of determining abnormal returns

Let us stack $u_{i,e+t}$ into $u_{e+t}$ for all stocks *i* at date $e+t$. Then, we postulate the model for $u_{e+t}$ in $t\in [-1,+10]$ as:

8 $$\begin{aligned} u_{e+t}= & {} \lambda u_{e+t-1} + \kappa W_{F_e} u_{e+t-1} + \beta _1 Tariff_{e+t,US} + \beta _2 Tariff_{e+t,CN} + \varepsilon _{e+t}.\nonumber \\ \end{aligned}$$

We can write this differently, after defining the $F_e\times F_e$ identity matrix $I_{F_e}$, as

9 $$\begin{aligned} u_{e+t}= & {} \left( \lambda I_{F_e} + \kappa W_{F_e}\right) u_{e+t-1} + \beta _1 Tariff_{e+t,US} + \beta _2 Tariff_{e+t,CN} + \varepsilon _{e+t},\qquad \end{aligned}$$

10 $$\begin{aligned}= & {} R_{e}u_{e+t-1} + \beta _1 Tariff_{e+t,US} + \beta _2 Tariff_{e+t,CN} + \varepsilon _{e+t}, \end{aligned}$$

11 $$\begin{aligned} R_{e}= & {} \left( \lambda I_{F_e} + \kappa W_{F_e}\right) . \end{aligned}$$

When allowing the parameters $(\lambda ,\kappa ,\beta _1,\beta _2)$ to be firm-specific,^5^ we obtain, after defining for the set of firms around event *e* as ${\mathfrak {N}}_e$ and the matrices $L_e=\mathrm{diag}_{i\in {\mathfrak {N}}_e}(\lambda _i)$, $S_{e}=\mathrm{diag}_{i\in {\mathfrak {N}}_e}(\kappa _i)$, $B_{1e}=\mathrm{diag}_{i\in {\mathfrak {N}}_e}(\beta _{1i})$, and $B_{2e}=\mathrm{diag}_{i\in {\mathfrak {N}}_e}(\beta _{2i})$:

12 $$\begin{aligned} u_{e+t}= & {} \left( L_{e} + S_{e}W_{F_e}\right) u_{e+t-1} + B_{1e} \mathrm{Tariff}_{e+t,US} + B_{2e} \mathrm{Tariff}_{e+t,CN} + \varepsilon _{e+t}, \qquad \end{aligned}$$

13 $$\begin{aligned}= & {} R_{e}u_{e+t-1} + B_{1e} \mathrm{Tariff}_{e+t,US} + B_{2e} \mathrm{Tariff}_{e+t,CN} + \varepsilon _{e+t}, \end{aligned}$$

14 $$\begin{aligned} R_{e}= & {} \left( L_{e} + S_{e}W_{F_e}\right) . \end{aligned}$$

Define $x_{e}=\beta _1 \mathrm{Tariff}_{e,US} + \beta _2 \mathrm{Tariff}_{e,CN}$ or $x_{e}=x=B_{1e} \mathrm{Tariff}_{e,US} + B_{2e} \mathrm{Tariff}_{e,CN}$ for all *e*, where $\mathrm{Tariff}_{e,US}$ and $\mathrm{Tariff}_{e,CN}$ are the tariffs set by the USA and China, respectively, on event date *e*. The latter will be sufficient, as we will consider only effects and experiments associated with $\mathrm{Tariff}_{e+t,US}$ and $\mathrm{Tariff}_{e+t,CN}$ which are nonzero on day $t=0$ and zero otherwise.

In order to gauge the transition and long-run effects of a particular shock $x_{e}$, we will let this process run, assuming that $x_{e}$ is absent (zero) for any time period prior to and after event time *e*, where $t=0$. Hence, the effects of the shock $x_{e}$ will fade with time, if the elements of $R_{e}$ are properly bounded.

As $E(\varepsilon _{e+t})=0$ for any *e* and *t*, what will be relevant is

15 $$\begin{aligned} E(u_{e+t})= & {} 0\text { for }t<0, \end{aligned}$$

16 $$\begin{aligned} E(u_{e+t})= & {} R^{t}_{e}x_{e}\text { for }t\ge 0. \end{aligned}$$

Hence, with appropriately bounded elements of $R_e$, the long-run effect from that series is $\sum ^\infty _{p=0}R^p_{e}x_{e}=(I_{F_e}-R_e)^{-1}x_{e}$.

We can then consider the deviations of the long-run predictions $\left( I_{F_e} - {\widehat{R}}_{e}\right) ^{-1}{\widehat{x}}_{e}$, where ${\widehat{x}}_{e}$ is estimated using firm-specific parameters $(\beta _{1i},\beta _{2i})$ and ${\widehat{R}}_{e}$ is estimated using firm-specific parameters $(\lambda _i,\kappa _i)$ from four more restrictive, alternative models: one where network effects are absent with $\kappa _i=0$ but all other parameters are firm-specific, $(\lambda _i,\beta _{1i},\beta _{2i})$; one where the parameters are estimated from a pooled model so that $(\lambda _i,\kappa _i,\beta _{1i},\beta _{2i})$ are not firm-specific but common to all firms, $(\lambda ,\kappa ,\beta _{1},\beta _{2})$; one where $\lambda =0$ and $\kappa =0$ for all firms and $(\beta _{1i},\beta _{2i})$ are firm-specific, and one where $\lambda =0$ and $\kappa =0$ and $(\beta _1,\beta _2)$ are assumed to be identical across all firms.

## Data

We use daily Chinese stock-market data in a window of 1 day before up until 10 days after each US–China “trade-war” tariff-announcement or—implementation event from Datastream.^6^ We consider 19 events in this estimation. For year 2018, we use: March 29, April 2, 3, and 4, June 15 and 16, July 6 and 10, August 1, 3, 7, 8, and 23, September 18 and 24, December 14. And for year 2019, we use: May 5, 10, and 13.

Note that as there is a time difference between the trading hours in the United States and China, we use the treatment day as to be $e+1$ in China whenever the treatment (a tariff change) was announced by the USA. However, whenever China announces a tariff change, the USA reacts on the same day, consistent with the time difference. And when the tariff change was announced on weekends, the corresponding event date in our analysis would be the first weekday after that day. So the event dates we consider in our analysis for Chinese tariff changes are March 29, April 2 and 4, June 18, July 6, August 3, 8, and 23, September 18 and 24, and December 14 in year 2018, and May 13 in year 2019. And for US tariff changes, the event dates are April 4, June 18, July 9 and 11, August 2, 8, and 24, and September 19 and 25 in year 2018 , and May 6 and 13 in year 2019.

We retrieve stock-return data on active companies listed on the Shanghai and Shenzhen Stock Exchanges and returns on the Morgan Stanley Capital International (MSCI) national equity index for China from Datastream. Data on nominal exchange rates are from the World Bank. The import-share-weighted nominal exchange rate of China is constructed using import shares of China with respective to 39 countries and territories in the World-Input-Output Database (WIOD).^7^ These data underly the dependent variable $r_{i,e+t}$ as well as the explanatory variables $m_{e+t}$ (for the MSCI index) and $ex_{e+t}$ used in the estimation of the abnormal returns, $u_{i,e+t}$.

We then obtain the lists of products on which “trade-war” tariffs were announced or imposed for each event from each country’s official website (see Egger and Zhu 2019). The USA and China report these tariffs by using HTS codes, which we convert to HS2017 6-digit product lines. We use concordance tables from the United Nations to convert 6-digit HS2017 codes first to 5-digit SITC rev. 3 codes and subsequently to ISIC rev. 3 4-digit codes. This is done, because firms in Datastream have between one and ten sector codes, while tariffs are levied on products rather than sectors. Given that we can map the sector classification of all listed firms in the data to the same (2-digit ISIC) sectoral classification, the “trade-war” tariff-change measures can be matched to each firm *i* around each event date *e* and day *t* around it. We weight the tariff changes by the operating income as a share of total operating income of each firm reported in datastream in up to ten sectors, so that tariff changes for firms in the same main sector can still vary between firms. The same sector-level operating income of the firms are used to compute the sector-level total operating income and the firm-specific share in it, when constructing the elements of $h_{FS}$.

We convert the tariff-change data from the 4-digit ISIC industry classification into the ISIC rev. 3 2-digit sector classification used by the WIOD table. The latter permits measuring the input-share-weighted abnormal returns $W_{F_e} u_{e+t-1}$. Specifically, for this we use the WIOD table for 2011 as released in 2013. The China–China block in this WIOD table is used as $O_{S}$ in the construction of the firm-sector-to-firm-sector input–output matrix $O_{FS}$ above.

The final sample includes 3540 stocks/firms across all events. In the estimation of the model parameters in Sect. 4, we winsorize the abnormal returns and the tariff-rate-change variables in the first permille and the 999th permille to avoid a disproportionate influence of extreme values in the data on the estimates. We summarize some descriptive statistics on stock-market returns and abnormal returns in Tables 1 and 2 and “trade-war” tariff changes in Table 3.

**Table 1:** Summary statistics of day-today log changes of individual stock returns ($r_{i,e}$) on each US-Chinese “tariff-war” event date considered

Event date	No. of firms	Mean	SD	Min	P25	P50	P75	Max
2018/03/29	3035	0.00875	0.01949	$-$0.10648	0.00000	0.00749	0.01681	0.10008
2018/04/02	3038	0.00511	0.02269	$-$0.10444	$-$0.00687	0.00000	0.01307	0.11123
2018/04/04	3041	$-$0.00746	0.02216	$-$0.18003	$-$0.01869	$-$0.00722	0.00000	0.10228
2018/06/18	3154	0.00001	0.00039	0.00000	0.00000	0.00000	0.00000	0.02151
2018/07/06	3178	0.00101	0.02364	$-$0.11478	$-$0.00816	0.00000	0.01258	0.10725
2018/07/09	3183	0.02352	0.02404	$-$0.50943	0.01399	0.02284	0.03279	0.10677
2018/07/11	3188	$-$0.03075	0.02518	$-$0.11778	$-$0.04499	$-$0.03226	$-$0.01754	0.10536
2018/08/02	3206	$-$0.03100	0.02803	$-$0.11538	$-$0.04763	$-$0.03410	$-$0.01681	0.10008
2018/08/03	3210	$-$0.01538	0.02558	$-$0.12516	$-$0.02691	$-$0.01408	0.00000	0.09716
2018/08/08	3215	$-$0.01331	0.02495	$-$0.10873	$-$0.02532	$-$0.01316	0.00000	0.10318
2018/08/23	3242	0.00131	0.01786	$-$0.11394	$-$0.00551	0.00000	0.00816	0.09937
2018/08/24	3245	$-$0.00307	0.02025	$-$0.12665	$-$0.01227	0.00000	0.00000	0.21869
2018/09/18	3278	0.01719	0.02014	$-$0.12136	0.00658	0.01575	0.02490	0.11778
2018/09/19	3281	0.01234	0.01844	$-$0.10725	0.00000	0.01058	0.01835	0.10697
2018/09/24	3283	$-$0.00001	0.00064	$-$0.02740	0.00000	0.00000	0.00000	0.00976
2018/09/25	3284	$-$0.00899	0.01760	$-$0.13103	$-$0.01770	$-$0.00998	0.00000	0.10178
2018/12/14	3380	$-$0.02990	0.02134	$-$0.11394	$-$0.04256	$-$0.03077	$-$0.01852	0.09423
2019/05/06	3522	$-$0.08091	0.03345	$-$0.13158	$-$0.10763	$-$0.08935	$-$0.06407	0.09716
2019/05/13	3528	$-$0.01483	0.02669	$-$0.11590	$-$0.02949	$-$0.01754	0.00000	0.09716

The number of firms/stocks listed in this table corresponds to $F_e$ in the text. P25, P50, and P75 refer to the 25th, the 50th, and the 75th percentile of the distribution within an event. SD, Min, and Max refer to the standard deviation, the minimum, and the maximum value in the data

**Table 2:** Summary statistics of abnormal returns (${\widehat{u}}_{i,e}$) in logs on each event date

Event date	No. of obs.	Mean	SD	Min	P25	P50	P75	Max
2018/03/29	3035	$-$0.00121	0.05505	$-$0.51581	$-$0.00551	0.00185	0.01206	1.28037
2018/04/02	3038	0.00710	0.05595	$-$0.52755	$-$0.00067	0.00929	0.02068	1.26140
2018/04/04	3041	0.00515	0.05460	$-$0.36450	$-$0.00453	0.00837	0.01853	1.28037
2018/06/18	3154	$-$0.00549	0.10193	$-$0.89472	$-$0.00203	0.00039	0.00220	1.28037
2018/07/06	3178	$-$0.01055	0.08630	$-$0.73339	$-$0.01737	$-$0.00482	0.00586	1.14674
2018/07/09	3183	0.00364	0.09281	$-$0.89472	$-$0.00337	0.00876	0.02038	1.18440
2018/07/11	3188	$-$0.02494	0.10072	$-$0.89472	$-$0.03705	$-$0.02183	$-$0.00728	1.28037
2018/08/02	3206	$-$0.02999	0.06301	$-$0.81947	$-$0.04122	$-$0.02464	$-$0.00712	0.42114
2018/08/03	3210	$-$0.02397	0.06250	$-$0.81079	$-$0.03075	$-$0.01422	0.00037	0.31265
2018/08/08	3215	$-$0.02289	0.06325	$-$0.82916	$-$0.02749	$-$0.01296	0.00135	0.12562
2018/08/23	3242	$-$0.00368	0.11275	$-$0.89472	$-$0.00496	0.00469	0.01129	1.28037
2018/08/24	3245	$-$0.01951	0.11005	$-$0.89472	$-$0.02063	$-$0.00692	$-$0.00105	1.28037
2018/09/18	3278	0.00466	0.14026	$-$0.89472	$-$0.00135	0.01030	0.02104	1.28037
2018/09/19	3281	$-$0.00328	0.15911	$-$0.89472	$-$0.01366	$-$0.00438	0.00744	1.28037
2018/09/24	3283	0.00183	0.15542	$-$0.89472	0.00004	0.00445	0.00765	1.28037
2018/09/25	3284	$-$0.01356	0.15856	$-$0.89472	$-$0.02372	$-$0.01351	$-$0.00218	1.28037
2018/12/14	3380	$-$0.02567	0.06070	$-$0.89472	$-$0.03953	$-$0.02435	$-$0.00909	0.40266
2019/05/06	3522	$-$0.07189	0.07928	$-$0.89472	$-$0.09278	$-$0.07496	$-$0.04856	1.18389
2019/05/13	3528	$-$0.00958	0.05953	$-$0.56751	$-$0.02391	$-$0.01025	0.00531	0.99335

The number of firms/stocks listed in this table corresponds to $F_e$ in the text. P25, P50, and P75 refer to the 25th, the 50th, and the 75th percentile of the distribution within an event. SD, Min, and Max refer to the standard deviation, the minimum, and the maximum value in the data

**Table 3:** Summary statistics of tariff changes in percent on each event date

Event date	No. of obs.	Mean	SD	Min	P25	P50	P75	Max
*Panel A. US tariff-change measures*								
2018/04/04	3041	10.28305	12.28423	0.00000	0.00000	0.00000	25.00000	29.81556
2018/06/18	3154	9.43003	12.09408	0.00000	0.00000	0.00000	25.00000	29.81556
2018/07/09	3183	6.61279	11.00388	0.00000	0.00000	0.00000	25.00000	29.81556
2018/07/11	3188	7.07010	4.86595	0.00000	0.00000	10.00000	10.00000	29.81556
2018/08/02	3206	17.41734	11.49074	0.00000	0.00000	25.00000	25.00000	29.81556
2018/08/08	3215	9.48427	12.10803	0.00000	0.00000	0.00000	25.00000	29.81556
2018/08/24	3245	9.53084	12.11781	0.00000	0.00000	0.00000	25.00000	29.81556
2018/09/19	3281	7.11132	4.83835	0.00000	0.00000	10.00000	10.00000	29.81556
2018/09/25	3284	7.11396	4.83692	0.00000	0.00000	10.00000	10.00000	29.81556
2019/05/06	3522	17.83259	11.29887	0.00000	0.00000	25.00000	25.00000	29.81556
2019/05/13	3528	17.83769	11.29647	0.00000	0.00000	25.00000	25.00000	29.81556
*Panel B. Chinese tariff-change measures*								
2018/03/29	3035	1.80514	5.28791	0.00000	0.00000	0.00000	0.00000	27.81664
2018/04/02	3038	1.80335	5.28560	0.00000	0.00000	0.00000	0.00000	27.81664
2018/04/04	3041	4.40556	9.47761	0.00000	0.00000	0.00000	0.00000	27.81664
2018/06/18	3154	10.41761	12.25239	0.00000	0.00000	0.00000	25.00000	27.81664
2018/07/06	3178	1.54234	5.98019	0.00000	0.00000	0.00000	0.00000	27.81664
2018/08/03	3210	10.81270	7.27262	0.00000	0.00000	15.00000	15.00000	27.81664
2018/08/08	3215	12.41985	12.44650	0.00000	0.00000	8.65231	25.00000	27.81664
2018/08/23	3242	12.44307	12.44518	0.00000	0.00000	9.14726	25.00000	27.81664
2018/09/18	3278	16.08322	10.58105	0.00000	0.00000	22.50000	22.50000	27.81664
2018/09/24	3283	16.09300	10.57595	0.00000	0.00000	22.50000	22.50000	27.81664
2018/12/14	3380	0.00083	0.03480	0.00000	0.00000	0.00000	0.00000	1.89987
2019/05/13	3528	11.04848	7.13713	0.00000	0.00000	15.00000	15.00000	27.81664

The number of firms/stocks listed in this table corresponds to $F_e$ in the text. P25, P50, and P75 refer to the 25th, the 50th, and the 75th percentile of the distribution of tariff-change measures adopted by the USA (Panel A) and China (Panel B) within an event. SD, Min, and Max refer to the standard deviation, the minimum, and the maximum value in the tariff-change data

First of all, the values of firm-/stock-specific stock-market returns, $r_{i,e}$, in Table 1 are small, because these values reflect day-to-day log changes in the stock prices. Clearly, we use more data than the ones reported in the table, as the summary statistics there only pertain to the very date when a tariff shock (by announcement or implementation) was realized. Recall that in order to estimate abnormal returns, $u_{i,e+t}$, we utilize data over an interval of about 250 trading days prior to any event. However, the table for just one day per event is still informative about the fact that there is a lot of variation in these returns, as the standard deviation of the returns on many event dates is larger than the average of these returns. Also the maximum and the interquartile ranges are quite large.

Table 2 suggests that when netting out the common factor associated with the market-return index (${\widehat{\zeta }}_{1i} m_{e}$) and import-share-weighted nominal exchanges (${\widehat{\zeta }}_{2i} ex_{e}$) after estimating Eq. (1) there is a tendency for the variation in abnormal returns (${\widehat{u}}_{i,e}$) to be larger relative to their mean than it was the case for stock-market returns ($r_{i,e}$) in Table 1. This is not surprising, as the regressions based on Eq. (1) use data up until the day $e-361$ prior to event *e*, and we would expect the variation in ${\widehat{u}}_{i,e+t}$ to increase as we move further forward from $e-361$ to time *e*.

Table 3 summarizes the tariff changes in percent on each event date. Recall that there is variation across Chinese firms in the tariff exposure for two reasons: First, the tariffs applied varied to a large extent across sectors (e.g., not all sectors were exposed to tariff increases, and the imposed tariffs were not identical across sectors where tariffs had been implemented); second, firms are active in various industries at different operating-income intensity so that they were exposed heterogeneously to the same tariffs schedule. In any case, this table suggests that there is a big variation in the average tariffs set between events, and also the variation within events between firms is large. The latter again shows in the standard deviation of tariff changes exceeding the mean in several events.

## Estimation results

In what follows, we will summarize the estimates of $(\lambda _i,\kappa _i,\beta _{1i},\beta _{2i})$ in the model where all these parameters are firm-specific. With a restrictive model, where $\lambda =0$ and $\kappa =0$ and $(\beta _{1i},\beta _{2i})$ are forced to be the same across all firms, we obtain ${\widehat{\beta }}_{1}=-0.00054686$ and ${\widehat{\beta }}_{2}=0.0002207$. In a slightly more flexible model, where $(\lambda _i,\kappa _i,\beta _{1i},\beta _{2i})$ may all be nonzero but the same across all firms, we obtain ${\widehat{\lambda }}=0.95247583$, and ${\widehat{\kappa }}=-1.0578604$, ${\widehat{\beta }}_{1}=-0.00059234$, and ${\widehat{\beta }}_{2}=0.00037713$.

**Table 4:** Summary statistics of the estimates of coefficients $(\lambda _i,\kappa _i,\beta ^{\prime }_i)$

Parameter	No. of firms	Mean	SD	Min	P25	P50	P75	Max
${\widehat{\lambda }}_i$	3540	0.13004	0.36399	$-$0.79097	$-$0.09593	0.02187	0.19302	1.07509
${\widehat{\kappa }}_i$	3540	$-$0.29111	0.56680	$-$3.12840	$-$0.42367	$-$0.15450	0.01151	5.85478
${\widehat{\beta }}_{1i}$	3540	$-$0.00201	0.06111	$-$3.31771	$-$0.00084	$-$0.00035	0.00000	0.83823
${\widehat{\beta }}_{2i}$	3540	0.00030	0.03279	$-$1.83927	0.00000	0.00016	0.00048	0.36993

The number of firms/stocks listed in this table corresponds to $F_e$ in the text. P25, P50, and P75 refer to the 25th, the 50th, and the 75th percentile of the distribution within an event. SD, Min, and Max refer to the standard deviation, the minimum, and the maximum value in the data

**Table 5:** *F*-tests of the firm-specific-parameter model with dynamic and network effects against restrictive alternatives

Null model	*F*-statistic	*P* value
*Panel A. Firm-specific versus constant parameters*		
$(\lambda _i,\kappa _i=0,\beta ^{\prime }_i)$	10.293	0.000
$(\lambda _i=\lambda ,\kappa _i=\kappa ,\beta ^{\prime }_i=\beta ^{\prime })$	27.520	0.000
$(\lambda _i=0,\kappa _i=0,\beta ^{\prime }_i)$	74.635	0.000
$(\lambda _i=0,\kappa _i=0,\beta ^{\prime }_i=\beta ^{\prime })$	137.943	0.000
*Panel B. Firm-specific versus sector-specific parameters*		
$(\lambda _{is}=\lambda _s,\kappa _{is}=0,\beta ^{\prime }_{is}=\beta ^{\prime }_s)$	36.076	0.000
$(\lambda _{is}=0,\kappa _{is}=0,\beta ^{\prime }_{is}=\beta ^{\prime }_s)$	137.678	0.000
$(\lambda _{is}=\lambda _s,\kappa _{is}=\kappa _s,\beta ^{\prime }_{is}=\beta ^{\prime }_s)$	25.625	0.000

The model under the null (Null model) indicates which parameters are restricted relative to the benchmark model (the Alternative model), where the parameters for firm *i*, $(\lambda _i,\kappa _i,\beta ^{\prime }_i)$ in Panel A and parameters for firm *i* in sector *s*, $(\lambda _{is},\kappa _{is},\beta ^{\prime }_{is})$ in Panel B are unrestricted. The *F*-statistic is the normalized difference in residual sums of squares of the respective model under the null and the benchmark model normalized by the difference in residual degrees of freedom relative to the benchmark model’s residual sum of squares normalized by its residual sum of squares

The estimates in Table 4 indicate that there is a large degree of variation in the respective parameters between firms. Even though we do not report standard errors on these coefficients, models with restrictions on these parameters to be common or zero are rejected against the proposed model with firm-specific parameters. As all considered more restrictive models are nested in the proposed, flexible one with firm-specific parameters, we can straightforwardly compare them statistically by way of F-statistics.

In Panel A of Table 5 we report on the F-statistics and the respective p values using event-specific data and appropriate residual and restrictions degrees of freedom for the three null models relative to the most flexible one, where $(\lambda _i,\kappa _i,\beta _{1i},\beta _{2i})$ are all firm-specific. The table indicates that every one of the considered restricted models is rejected at a high level of statistical significance against the flexible alternative model. Hence, serial correlation within and network correlation between the firms should not be ignored. Note in particular that the null model at the bottom of Table 5, which only restricts network effects to be absent but allows $(\lambda _i,\beta ^{\prime }_i)$ to be firm-specific is rejected against the model which also considers firm-specific network effects in abnormal returns ($\kappa _i\ne 0$). Some of the individual parameters ${\widehat{\lambda }}_i$ and ${\widehat{\kappa }}_i$ are quite large in absolute value. However, we should not conclude from this that the model is not stable. What matters is that $(I_{F_e}-{\widehat{R}}_e)^{-1}$ exists, as is the case, here.

In Panel B of Table 5 we report results on the *F*-tests of the firm-specific parameters model against ones with parameters that are common across sectors and event windows. Clearly, the sector-specific-parameters models are rejected against the one where all parameters are firm-specific. The sector-specific parameter densities overlap with the firm-specific ones, but the variance in the firm-specific parameters is much bigger, indicating that there is a lot of heterogeneity in the responses across firms.

**Table 6:** Effects of “trade-war” tariff shocks when all parameters are firm-specific

Event date	No. of obs.	Mean	SD	Min	P25	P50	P75	Max
*Panel A. Short-run effects* ($x_{e}$)								
2018/03/29	3035	0.00056	0.00243	$-$0.02919	0.00000	0.00000	0.00000	0.02361
2018/04/02	3038	0.00056	0.00243	$-$0.02919	0.00000	0.00000	0.00000	0.02361
2018/04/04	3041	$-$0.00259	0.01036	$-$0.10162	$-$0.00526	0.00000	0.00000	0.32597
2018/06/18	3154	$-$0.00285	0.11159	$-$6.24498	$-$0.00360	0.00000	0.00000	0.24213
2018/07/06	3178	0.00056	0.00314	$-$0.03649	0.00000	0.00000	0.00000	0.05118
2018/07/09	3183	$-$0.00262	0.00653	$-$0.04925	0.00000	0.00000	0.00000	0.04728
2018/07/11	3188	$-$0.00615	0.10874	$-$6.10375	$-$0.00712	$-$0.00312	0.00000	0.16174
2018/08/02	3206	$-$0.01538	0.27108	$-$15.25938	$-$0.01787	$-$0.00782	0.00000	0.40435
2018/08/03	3210	0.00366	0.02539	$-$0.22219	0.00000	0.00158	0.00583	0.98330
2018/08/08	3215	$-$0.00195	0.11048	$-$6.24498	$-$0.00108	0.00000	0.00000	0.24213
2018/08/23	3242	0.00242	0.11369	$-$6.24498	0.00000	0.00000	0.00702	1.62620
2018/08/24	3245	$-$0.00433	0.02893	$-$1.54361	$-$0.00721	0.00000	0.00000	0.35296
2018/09/18	3278	0.00562	0.04031	$-$0.33328	0.00000	0.00244	0.00887	1.67591
2018/09/19	3281	$-$0.00615	0.10720	$-$6.10375	$-$0.00729	$-$0.00315	0.00000	0.16174
2018/09/24	3283	0.00565	0.04029	$-$0.33328	0.00000	0.00247	0.00891	1.67591
2018/09/25	3284	$-$0.00615	0.10715	$-$6.10375	$-$0.00730	$-$0.00316	0.00000	0.16174
2018/12/14	3380	0.00000	0.00039	$-$0.01637	0.00000	0.00000	0.00000	0.01448
2019/05/06	3522	$-$0.01908	0.25929	$-$15.25938	$-$0.02061	$-$0.00873	0.00000	0.40435
2019/05/13	3528	$-$0.01074	0.24094	$-$14.28088	$-$0.01274	$-$0.00347	0.00000	0.18216
*Panel B. Long-run effects* ($(I_{F_e}-R_e)^{-1}x_{e}$)								
2018/03/29	3035	0.00046	0.00441	$-$0.02773	$-$0.00030	$-$0.00006	0.00007	0.14019
2018/04/02	3038	0.00045	0.00441	$-$0.02773	$-$0.00030	$-$0.00006	0.00007	0.14019
2018/04/04	3041	$-$0.00213	0.02049	$-$0.27313	$-$0.00406	0.00000	0.00159	0.52109
2018/06/18	3154	$-$0.00064	0.15806	$-$8.68956	$-$0.00270	0.00011	0.00308	0.93491
2018/07/06	3178	0.00038	0.00902	$-$0.02270	$-$0.00036	$-$0.00009	0.00004	0.32088
2018/07/09	3183	$-$0.00250	0.02313	$-$0.36047	$-$0.00123	0.00004	0.00099	0.23659
2018/07/11	3188	$-$0.00435	0.15158	$-$8.49319	$-$0.00587	$-$0.00145	0.00177	0.23979
2018/08/02	3206	$-$0.01085	0.37811	$-$21.23296	$-$0.01480	$-$0.00361	0.00447	0.59899
2018/08/03	3210	0.00474	0.03346	$-$0.17001	$-$0.00104	0.00106	0.00512	1.36161
2018/08/08	3215	0.00065	0.15577	$-$8.68955	$-$0.00123	0.00026	0.00394	0.39084
2018/08/23	3242	0.00557	0.15769	$-$8.68943	$-$0.00112	0.00059	0.00723	0.73194
2018/08/24	3245	$-$0.00450	0.03884	$-$0.69529	$-$0.00488	0.00000	0.00153	0.56464
2018/09/18	3278	0.00770	0.05688	$-$0.33733	$-$0.00169	0.00162	0.00778	2.33206
2018/09/19	3281	$-$0.00385	0.15025	$-$8.49319	$-$0.00601	$-$0.00142	0.00189	0.27158
2018/09/24	3283	0.00788	0.05726	$-$0.33793	$-$0.00171	0.00161	0.00780	2.33206
2018/09/25	3284	$-$0.00386	0.15020	$-$8.49319	$-$0.00602	$-$0.00144	0.00190	0.27158
2018/12/14	3380	0.00002	0.00058	$-$0.02530	0.00000	0.00000	0.00002	0.02019
2019/05/06	3522	$-$0.02313	0.37174	$-$21.23300	$-$0.01752	$-$0.00421	0.00527	0.67987
2019/05/13	3528	0.00114	0.34370	$-$19.87135	$-$0.01076	$-$0.00068	0.00745	0.92391

The number of firms/stocks listed in this table corresponds to $F_e$ in the text. P25, P50, and P75 refer to the 25th, the 50th, and the 75th percentile of the distribution within an event. SD, Min, and Max refer to the standard deviation, the minimum, and the maximum value in the data

**Table 7:** Direct effects of shocks when parameters are identical between firms and both dynamic and network effects are absent (short-run=long-run; $(I_{F_e}-R_e)^{-1}x_{e}=x_{e}$)

Event date	No. of obs.	Mean	SD	Min	P25	P50	P75	Max
2018/03/29	3035	0.00032	0.00399	$-$0.20843	0.00000	0.00000	0.00000	0.01049
2018/04/02	3038	0.00032	0.00399	$-$0.20843	0.00000	0.00000	0.00000	0.01049
2018/04/04	3041	$-$0.00416	0.05062	$-$1.02455	$-$0.01367	0.00000	0.00000	2.54680
2018/06/18	3154	$-$0.01305	0.59996	$-$33.58134	$-$0.00815	0.00000	0.00000	2.54680
2018/07/06	3178	0.00031	0.00195	$-$0.07450	0.00000	0.00000	0.00000	0.01080
2018/07/09	3183	$-$0.00287	0.04702	$-$0.10548	$-$0.01367	0.00000	0.00000	2.62130
2018/07/11	3188	$-$0.00878	0.28576	$-$16.08817	$-$0.00547	$-$0.00547	0.00000	1.04852
2018/08/02	3206	$-$0.02188	0.71238	$-$40.22041	$-$0.01367	$-$0.01367	0.00000	2.62130
2018/08/03	3210	0.00395	0.09392	$-$0.68106	0.00000	0.00331	0.00331	5.26170
2018/08/08	3215	$-$0.01273	0.59317	$-$33.58134	$-$0.00815	0.00000	0.00000	1.56340
2018/08/23	3242	$-$0.00772	0.59026	$-$33.58134	0.00000	0.00202	0.00552	0.69328
2018/08/24	3245	$-$0.00495	0.05587	$-$1.71783	$-$0.01367	0.00000	0.00000	2.62130
2018/09/18	3278	0.00622	0.15874	$-$1.01000	0.00000	0.00497	0.00497	9.01193
2018/09/19	3281	$-$0.00866	0.28168	$-$16.08817	$-$0.00547	$-$0.00547	0.00000	1.04852
2018/09/24	3283	0.00621	0.15862	$-$1.01000	0.00000	0.00497	0.00497	9.01193
2018/09/25	3284	$-$0.00866	0.28155	$-$16.08817	$-$0.00547	$-$0.00547	0.00000	1.04852
2018/12/14	3380	$-$0.00001	0.00018	$-$0.00331	0.00000	0.00000	0.00000	0.00042
2019/05/06	3522	$-$0.02100	0.67967	$-$40.22041	$-$0.01367	$-$0.01367	0.00000	2.62130
2019/05/13	3528	$-$0.01713	0.58979	$-$34.95871	$-$0.01036	$-$0.01036	0.00000	1.94024

The number of firms/stocks listed in this table corresponds to $F_e$ in the text. P25, P50, and P75 refer to the 25th, the 50th, and the 75th percentile of the distribution within an event. SD, Min, and Max refer to the standard deviation, the minimum, and the maximum value in the data

Tables 6 and 7 focus on a summary of the resulting effect estimates. In the proposed model which allows all parameters $(\lambda _i,\kappa _i,\beta ^{\prime }_{i})$ to vary across firms, the effects materializing instantly on event date *e* are ${\widehat{x}}_e$, where the latter is estimated based on firm-specific parameters on tariff changes, $\beta _{i}$. The corresponding results are summarized in Panel A of Table 6. In the long run, after all dynamic and rippling effects from this shock have faded, the cumulative effect of the tariff change (in absence of any further shocks in subsequent days) would be $(I_{F_e}-{\widehat{R}}_e)^{-1}{\widehat{x}}_e$. The corresponding results are summarized in Panel B of Table 6. The findings of Table 6 are contrasted with the ones of a customary model in Table 7, where $(\beta ^{\prime }_{i} =\beta )$ is assumed to be common across all firms and both dynamic and network-rippling effects are assumed to be absent, $(\lambda _i=0,\kappa _i=0)$.

A comparison of the reported moments of the distributions of the effects indicates that the long-run effects tend to be larger than their short-run counterparts. Hence, there is dynamic (individual plus network) amplification and accumulation of the effects of tariff shocks on abnormal returns, according to Table 6. Moreover, a consultation of the moments of the effects distribution in Table 7, where dynamic and network effects are assumed to be absent $(\lambda _i=0,\kappa _i=0)$ and immediate tariff-shock effects are assumed to be common across firms $(\beta ^{\prime }_{i} =\beta )$ indicates that the bias in the results is quite substantial, when ignoring response heterogeneity and dynamic individual and network adjustment.

Towards a quantitative interpretation of the results, note that the effects, e.g., in Table 6 can be contrasted with the numbers on abnormal returns (in logs) in Table 2. This comparison suggests the following insights. First, the average effects in Table 6 are smaller by roughly one order of magnitude than the average abnormal returns in logs. However, these average responses are driven by effects on directly exposed firms in China. Note that the effects at the median (P50) in Table 6 are small. Hence, most firms receive shocks only through the input–output network, but this indirect exposure is modest for the median firm. Firms which are directly exposed to shocks or closely related to ones in targeted sectors receive bigger shocks (see the reported changes in the tails of the distribution in Table 6).

**Table 8:** Effects of “trade-war” tariff shocks when ARs are estimated with the Fama-French SMB and HML factors included in the model and all parameters are firm-specific

Event date	No. of obs.	Mean	SD	Min	P25	P50	P75	Max
*Panel A. Short-run effects* ($x_{e}$)								
2018/03/29	2800	0.00014	0.00199	$-$0.03297	0.00000	0.00000	0.00000	0.01868
2018/04/02	2801	0.00016	0.00203	$-$0.03297	0.00000	0.00000	0.00000	0.01890
2018/04/04	2790	$-$0.00179	0.01550	$-$0.05539	$-$0.00354	0.00000	0.00000	0.66582
2018/06/18	2787	0.04626	2.50270	$-$0.10063	$-$0.00339	0.00000	0.00000	132.11870
2018/07/06	2779	0.00016	0.00224	$-$0.04121	0.00000	0.00000	0.00000	0.03114
2018/07/09	2784	$-$0.00132	0.00489	$-$0.03689	0.00000	0.00000	0.00000	0.02995
2018/07/11	2791	$-$0.00283	0.01839	$-$0.04554	$-$0.00591	$-$0.00160	0.00000	0.92715
2018/08/02	2790	$-$0.00711	0.04575	$-$0.11386	$-$0.01483	$-$0.00402	0.00000	2.31788
2018/08/03	2790	$-$0.00629	0.39209	$-$20.70106	$-$0.00103	0.00000	0.00377	0.47091
2018/08/08	2784	0.04623	2.50405	$-$0.10773	$-$0.00331	0.00000	0.00000	132.11870
2018/08/23	2804	0.04830	2.49506	$-$0.08656	0.00000	0.00000	0.00177	132.11870
2018/08/24	2787	$-$0.00241	0.00816	$-$0.11298	$-$0.00289	0.00000	0.00000	0.21340
2018/09/18	2797	$-$0.01078	0.67064	$-$35.45555	$-$0.00137	0.00000	0.00587	0.70636
2018/09/19	2802	$-$0.00281	0.01833	$-$0.04554	$-$0.00592	$-$0.00155	0.00000	0.92715
2018/09/24	2810	$-$0.01081	0.66909	$-$35.45555	$-$0.00141	0.00000	0.00574	0.70636
2018/09/25	2786	$-$0.00286	0.01836	$-$0.04554	$-$0.00592	$-$0.00156	0.00000	0.92715
2018/12/14	2804	0.00000	0.00027	$-$0.00596	0.00000	0.00000	0.00000	0.01289
2019/05/06	2792	$-$0.00735	0.04580	$-$0.11386	$-$0.01512	$-$0.00410	0.00000	2.31788
2019/05/13	2778	$-$0.01337	0.34893	$-$18.38318	$-$0.01311	$-$0.00266	0.00000	0.35705
*Panel B. Long-run effects* ($(I_{F_e}-R_e)^{-1}x_{e}$)								
2018/03/29	2800	0.00014	0.00216	$-$0.02630	$-$0.00013	0.00000	0.00016	0.01838
2018/04/02	2801	0.00017	0.00222	$-$0.02631	$-$0.00014	0.00000	0.00017	0.01985
2018/04/04	2790	$-$0.00170	0.02077	$-$0.07025	$-$0.00467	$-$0.00056	0.00115	0.70542
2018/06/18	2787	0.04934	2.61984	$-$0.56925	$-$0.00916	$-$0.00037	0.00477	138.23220
2018/07/06	2779	0.00017	0.00240	$-$0.03249	$-$0.00014	0.00000	0.00016	0.03804
2018/07/09	2784	$-$0.00144	0.00591	$-$0.03574	$-$0.00208	$-$0.00005	0.00049	0.04160
2018/07/11	2791	$-$0.00305	0.01977	$-$0.05334	$-$0.00693	$-$0.00247	0.00066	0.97091
2018/08/02	2790	$-$0.00761	0.04903	$-$0.13340	$-$0.01718	$-$0.00622	0.00140	2.42733
2018/08/03	2790	$-$0.00664	0.41038	$-$21.65892	$-$0.00281	0.00005	0.00480	0.43244
2018/08/08	2784	0.04875	2.62114	$-$0.82177	$-$0.00894	$-$0.00018	0.00517	138.23230
2018/08/23	2804	0.05100	2.61179	$-$0.78156	$-$0.00556	0.00001	0.00686	138.23170
2018/08/24	2787	$-$0.00254	0.00956	$-$0.11990	$-$0.00497	$-$0.00048	0.00087	0.21239
2018/09/18	2797	$-$0.01133	0.70194	$-$37.09612	$-$0.00424	0.00013	0.00759	0.63491
2018/09/19	2802	$-$0.00301	0.01997	$-$0.08890	$-$0.00695	$-$0.00248	0.00064	0.97091
2018/09/24	2810	$-$0.01127	0.70032	$-$37.09613	$-$0.00424	0.00007	0.00759	0.63466
2018/09/25	2786	$-$0.00318	0.02003	$-$0.18374	$-$0.00688	$-$0.00248	0.00054	0.97093
2018/12/14	2804	0.00000	0.00037	$-$0.00497	$-$0.00001	0.00000	0.00001	0.01837
2019/05/06	2792	$-$0.00799	0.04930	$-$0.30065	$-$0.01752	$-$0.00639	0.00121	2.42734
2019/05/13	2778	$-$0.01429	0.36546	$-$19.23157	$-$0.01867	$-$0.00544	0.00360	0.29653

The number of firms/stocks listed in this table corresponds to $F_e$ in the text. P25, P50, and P75 refer to the 25th, the 50th, and the 75th percentile of the distribution within an event. SD, Min, and Max refer to the standard deviation, the minimum, and the maximum value in the data

**Table 9:** *F*-tests for the firm-specific parameter model against restrictive alternatives when the Fama-French SMB and HML factors are further included in the AR estimation model

Null model	*F*-statistic	*P* value
$(\lambda _i,\kappa _i=0,\beta ^{\prime }_i)$	1.065	0.008
$(\lambda _i=\lambda ,\kappa _i=\kappa ,\beta ^{\prime }_i=\beta ^{\prime })$	1.368	0.000
$(\lambda _i=0,\kappa _i=0,\beta ^{\prime }_i)$	1.053	0.003
$(\lambda _i=0,\kappa _i=0,\beta ^{\prime }_i=\beta ^{\prime })$	1.054	0.000

The model under the null (null model) indicates which parameters are restricted relative to the benchmark model (the Alternative model), where the parameters $(\lambda _i,\kappa _i,\beta ^{\prime }_i)$ are unrestricted. The F-statistic is the normalized difference in residual sums of squares of the respective model under the null and the benchmark model normalized by the difference in residual degrees of freedom relative to the benchmark model’s residual sum of squares normalized by its residual sum of squares

**Table 10:** Effects of “trade-war” tariff shocks when the first-to-third-order autocorrelations are accounted for in the model and all parameters are firm-specific

Event date	No. of obs.	Mean	SD	Min	P25	P50	P75	Max
*Panel A. Short-run effects* ($x_{e}$)								
2018/03/29	3035	0.00074	0.00378	$-$0.02178	0.00000	0.00000	0.00000	0.05969
2018/04/02	3038	0.00074	0.00378	$-$0.02178	0.00000	0.00000	0.00000	0.05969
2018/04/04	3041	$-$0.00037	0.03655	$-$1.83388	0.00000	0.00000	0.00035	0.05957
2018/06/18	3154	$-$0.01064	0.65657	$-$36.81770	0.00000	0.00000	0.00348	0.10447
2018/07/06	3178	0.00083	0.00439	$-$0.01969	0.00000	0.00000	0.00000	0.05957
2018/07/09	3183	$-$0.00102	0.00762	$-$0.07566	0.00000	0.00000	0.00000	0.06522
2018/07/11	3188	0.00162	0.13420	$-$0.84294	$-$0.00279	0.00000	0.00115	7.51717
2018/08/02	3206	0.00406	0.33456	$-$2.10734	$-$0.00700	0.00000	0.00291	18.79292
2018/08/03	3210	0.00445	0.10262	$-$0.52695	0.00000	0.00000	0.00573	5.76879
2018/08/08	3215	$-$0.00897	0.65034	$-$36.81770	0.00000	0.00000	0.00534	0.13030
2018/08/23	3242	$-$0.00723	0.64683	$-$36.81770	0.00000	0.00000	0.00618	0.25722
2018/08/24	3245	$-$0.00171	0.03910	$-$2.09110	0.00000	0.00000	0.00000	0.07144
2018/09/18	3278	0.00729	0.17368	$-$0.79042	0.00000	0.00000	0.00890	9.88045
2018/09/19	3281	0.00139	0.13231	$-$0.84294	$-$0.00291	0.00000	0.00114	7.51717
2018/09/24	3283	0.00728	0.17354	$-$0.79042	0.00000	0.00000	0.00888	9.88045
2018/09/25	3284	0.00140	0.13225	$-$0.84294	$-$0.00291	0.00000	0.00116	7.51717
2018/12/14	3380	$-$0.00001	0.00031	$-$0.01728	0.00000	0.00000	0.00000	0.00041
2019/05/06	3522	0.00295	0.31943	$-$2.10734	$-$0.00773	0.00000	0.00347	18.79292
2019/05/13	3528	0.00720	0.41518	$-$1.95181	$-$0.00397	0.00000	0.00627	24.56171
*Panel B. Long-run effects*								
2018/03/29	3035	0.00055	0.00483	$-$0.04446	$-$0.00019	$-$0.00004	0.00003	0.10893
2018/04/02	3038	0.00054	0.00483	$-$0.04446	$-$0.00019	$-$0.00004	0.00003	0.10893
2018/04/04	3041	$-$0.00147	0.04796	$-$2.20843	$-$0.00059	0.00005	0.00199	0.20945
2018/06/18	3154	$-$0.00165	0.80376	$-$44.65056	$-$0.00285	0.00010	0.00754	2.02500
2018/07/06	3178	0.00057	0.00638	$-$0.05568	$-$0.00018	$-$0.00004	0.00002	0.14769
2018/07/09	3183	$-$0.00125	0.02410	$-$0.36467	$-$0.00012	0.00001	0.00039	0.32550
2018/07/11	3188	$-$0.00024	0.16456	$-$1.02150	$-$0.00383	$-$0.00015	0.00148	9.11627
2018/08/02	3206	$-$0.00121	0.41236	$-$2.55757	$-$0.00963	$-$0.00037	0.00373	22.79241
2018/08/03	3210	0.00451	0.12675	$-$0.44670	$-$0.00234	0.00001	0.00494	6.99589
2018/08/08	3215	0.00338	0.79718	$-$44.65062	$-$0.00252	0.00039	0.00971	2.02658
2018/08/23	3242	0.00719	0.79456	$-$44.65319	$-$0.00272	0.00047	0.01077	1.92391
2018/08/24	3245	$-$0.00349	0.06799	$-$2.51795	$-$0.00043	0.00001	0.00098	0.73382
2018/09/18	3278	0.00883	0.22160	$-$0.76748	$-$0.00414	0.00000	0.00760	11.98304
2018/09/19	3281	$-$0.00239	0.16443	$-$1.02149	$-$0.00394	$-$0.00016	0.00165	9.11628
2018/09/24	3283	0.00877	0.22144	$-$0.76740	$-$0.00415	0.00000	0.00759	11.98304
2018/09/25	3284	$-$0.00231	0.16442	$-$1.02148	$-$0.00397	$-$0.00015	0.00165	9.11628
2018/12/14	3380	0.00000	0.00040	$-$0.02227	0.00000	0.00000	0.00001	0.00080
2019/05/06	3522	$-$0.00349	0.39018	$-$2.45915	$-$0.01046	$-$0.00044	0.00418	22.66496
2019/05/13	3528	$-$0.00066	0.50653	$-$2.47842	$-$0.00825	0.00000	0.00577	29.62224

The number of firms/stocks listed in this table corresponds to $F_e$ in the text. P25, P50, and P75 refer to the 25th, the 50th, and the 75th percentile of the distribution within an event. SD, Min, and Max refer to the standard deviation, the minimum, and the maximum value in the data

**Table 11:** *F*-tests for the firm-specific parameter model against restrictive alternatives when the first-order–third-order autocorrelations are accounted for in the model

Null model	*F*-statistic	*P*-value
$(\lambda _i,\kappa _i=0,\beta ^{\prime }_i)$	3.237	0.000
$(\lambda _i=\lambda ,\kappa _i=\kappa ,\beta ^{\prime }_i=\beta ^{\prime })$	7.150	0.000
$(\lambda _i=0,\kappa _i=0,\beta ^{\prime }_i)$	30.373	0.000
$(\lambda _i=0,\kappa _i=0,\beta ^{\prime }_i=\beta ^{\prime })$	59.539	0.000

The model under the null (null model) indicates which parameters are restricted relative to the benchmark model (the alternative model), where the parameters $(\lambda _i,\kappa _i,\beta ^{\prime }_i)$ are unrestricted. The F-statistic is the normalized difference in residual sums of squares of the respective model under the null and the benchmark model normalized by the difference in residual degrees of freedom relative to the benchmark model’s residual sum of squares normalized by its residual sum of squares

In Tables 8 and 9, we present the effect estimates and associated tests of a model which accounts for the *small minus big market capitalization (SMB)* and the *high minus low book-to-market value ratio (HML)* proposed by Fama and French (1992, 1993) on top of the factors included otherwise in the first-step stock-market-returns model. The results in Table 8 suggest that the magnitudes of tariff-war shock effects are very similar to the model which excludes the SMB and HML factors, and the F tests in Table 9 support the same conclusions as drawn from the comparable tests in the benchmark model of Table 5.

In Tables 10 and 11, we summarize the results based on a third-order-lag model rather than a first-order lag one as used in the benchmark analysis.^8^ The magnitudes of the short-run responses tend to be somewhat smaller on average while those of the long-run responses are somewhat larger in Table 10 than in Table 6. However, the range of the estimates is similar in broad terms in Table 10 to the ones reported in Table 6. The tests in Table 11 suggest that a firm-specific parameters model is supported relative to its more restrictive alternatives as before.

## Potential sources of endogeneity

Regarding the estimation the model in Eq. (12), we can think of two sources of endogeneity: the one of the network connections between firms through $W_{F_e}$, and the one through the presence of the lagged endogenous regressor $u_{e+t-1}$. However, the latter is a residual obtained from earlier firm-specific regressions, so that we ignore the latter source of endogeneity and focus on the former one in this section.

Note that the network matrix $W_{F_e}$ depends on the operating-income-share distribution across firms referred to as $h_{FS}$ above (for brevity, we will refer to operating income by sales in what follows). The latter is up to each firm *i*’s choice, which establishes an endogeneity concern, since the latter choice of firms, even though cast in the past, may be correlated with unobservables in the *i*th element of $\varepsilon _{e+t}$ in (8). Then, the term $\kappa W_{F_e} u_{e+t-1}$ would be endogenous and $\kappa$ might be biased.

In this section, we address this concern as follows. Let us introduce the following definitions: $\hbox {sales}_{{is}}$ are the sales of firm *i* in sector *s*; $SALES_s=\sum ^N_{i=1}\text {sales}_{is}$ are the aggregate sales of all firms in our data in sector *s*; ${\mathcal {N}}_i$ is the set of firms, whose sales shares are the biggest in the same sector as that of *i* but excluding firm *i*; $N_i$ is the number of firms (elements) in ${\mathcal {N}}_i$; $SALES_{is}=\sum _{j\in {\mathcal {N}}_i}\text {sales}_{js}$ are the aggregate sales of all firms except *i* in the set ${\mathcal {N}}_i$ in any sector *s*; ${\overline{SALES}}_{is}=\text {SALES}_{is}/N_{i}$ are the average sales of firms in ${\mathcal {N}}_i$ (which excludes *i*) in sector *s*. Then, we can define ${\widehat{h}}_{is}=\frac{\overline{\text {SALES}}_{is}}{\text {SALES}_s}$ for each firm *i* and sector *s* instead of $h_{is}$. This allows us to define ${\widehat{H}}_{FS}={\widehat{h}}_{FS}{\widehat{h}}^{\prime }_{FS}$, ${\widehat{M}}_{FS} = {\widehat{H}}_{FS} \circ (J_F\otimes O_S)$, ${\widehat{O}}_{FS} = {\widehat{M}}_{FS} \otimes K_{FS}$, and ${\widehat{O}}_F = Z^{\prime }_{FS}{\widehat{O}}_{FS}Z_{FS}$ as alternatives to the terms defined in Sect. 3. Using ${\widehat{O}}_F$ instead of $O_F$ in the definition of $W_{Fe}$, we can define a matrix ${\widehat{W}}_{Fe}$. The latter can serve to define ${\widehat{W}}_{Fe}u_{e+t-1}$ which can be used as an instrumental variable for $W_{Fe}u_{e+t-1}$ when estimating Eq. (8).

**Fig. 1:**
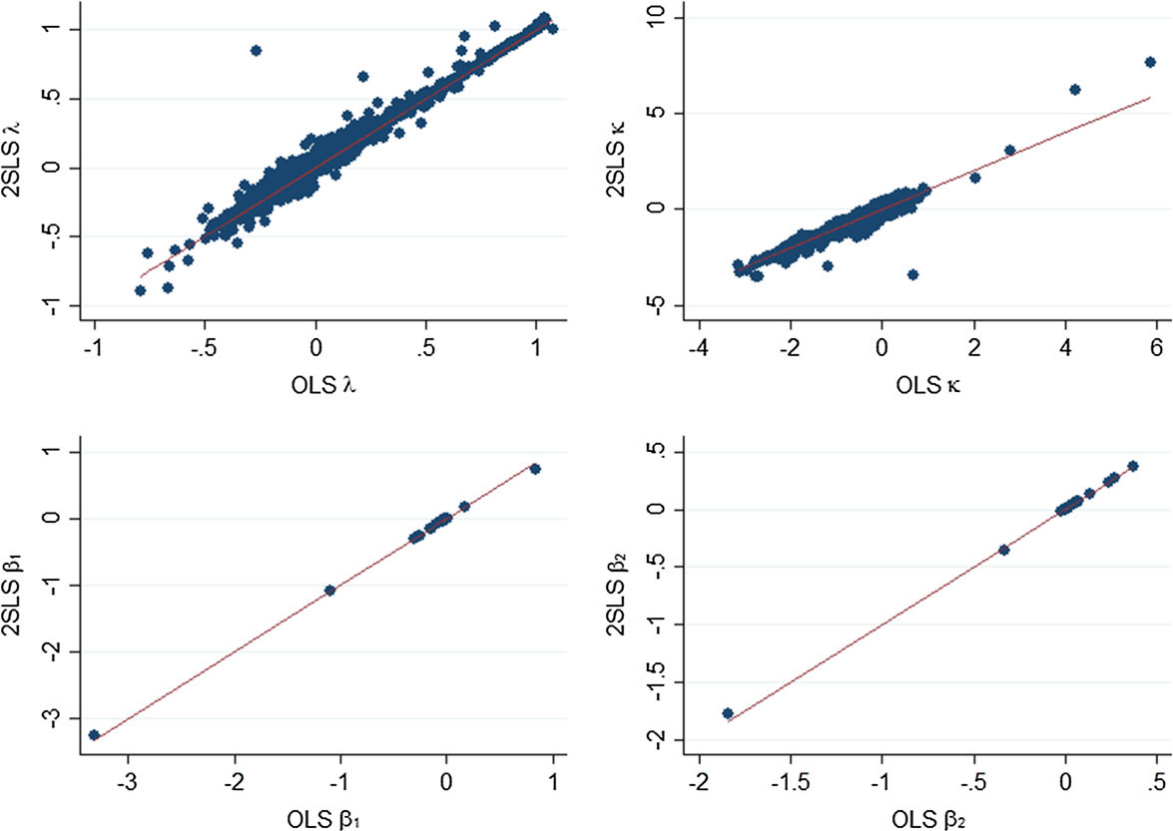
OLS estimates against 2SLS estimates Notes: This figure plots estimates of the parameters $\{\lambda ,\kappa ,\beta _1,\beta _2\}$ based on 2SLS against ones based on OLS

Doing so suggests that the instrument is valid (suggested by the associated weak-instrument test statistics). However, a comparison of the OLS and two-stage least-squares (2SLS) parameters suggests that the bias associated with the addressed endogeneity of $W_{F_e}$ is relatively minor. This can be seen from an inspection of Fig. 1, where we plot estimates of the parameters $\{\lambda ,\kappa ,\beta _1,\beta _2\}$ based on 2SLS against ones based on OLS. By and large and with a few exceptions, the two sets of parameters are well aligned around the $45^{\circ }$ line.

## Conclusions

This paper contributes to the literature which aims at estimating the effects of trade liberalization, trade deliberalization or other domestic or foreign shocks on the returns to individual stocks at stock markets. Related work tends to invoke three assumptions: (i) that the parameters on shocks which determine abnormal stock-market returns can be pooled across stocks/firms; (ii) that either adjustment costs are absent or at least common to all firms and small enough so that long-run effects of shocks on abnormal returns can be estimated from relatively small windows of time; and (iii) that shocks feature idiosyncratic effects on abnormal stock-market returns so that network effects among stocks are absent.

The present paper abandons these assumptions by allowing the process of abnormal stock-market returns to be governed by firm-specific parameters and by an autoregressive process which accounts for individual autocorrelation as well as dynamic adjustment through network effects. The paper then employs a large data set of daily stock-market returns in 2018 and 2019 to estimate abnormal returns and then determine these abnormal returns as a function of three ingredients, all of which may carry stock-specific parameters: shocks as reflected in trade-war tariff announcements or implementations by the USA and China, own lagged abnormal returns of a stock, and network-weighted abnormal returns by a stock. The paper does so by postulating network effects to emanate from input–output relationships between stock-market listed firms about whom the operating revenue structure is known. The latter is done by establishing a listed-firm–listed-firm input–output matrix based on the revenue structure of firms across sectors as well as the input–output table between sectors in China.

The results suggest that all three customary assumptions are rejected by the data at hand: the process of abnormal returns appears to call for firm-specific parameters; there is evidence of idiosyncratic adjustment costs; own lagged abnormal returns display a large variance in their importance for current stocks’ abnormal returns; and network effects among stocks contribute another important element in the variance of abnormal stock returns whose importance again varies largely between stocks.

We believe that the explicit consideration of adjustment costs and, particularly, of network effects could be an important future avenue of work. Network effects lead to nonlinear interactions among stocks, and shocks can have interesting and nontrivial effects on stock markets, an issue which is of potentially great importance when considering the vulnerability or resilience of stock markets and their dependence on core players in the network. In particular, it might be interesting to consider more flexible, multiplex networks in affecting stock markets than considered here.

## Footnotes

1 This is done to ensure that the end of the estimation window for the last event considered in this study—namely, the one on May 13, 2019—is December 31, 2017, which is prior to the first event of our analysis. Note that it is customary in the finance literature exploiting treatment effects at stock markets in event-type studies to use a number of days covering about one trading year to estimate the parameters used to determine abnormal returns at times after that year and a much shorter time window afterwards to estimate effects of certain events on those abnormal returns afterwards. The second window is kept short to ensure that the effects of events can be measured cleanly without much influence of other shocks than the interesting one.

2 This makes use of the notation introduced in Baltagi (2008).

3 Note that by the design of this matrix we consider network effects among all firms in the data, not just ones in the sectors that additional tariffs are (expected to) imposed on. Hence, the tariff shocks considered exert direct effects on firms in the target sector in China, but effects of these shocks may dissipate elsewhere through the input–output network.

4 Note that it is customary in input–output analysis to use such normalized matrices. In general, in network analysis, row normalization is one but not the only choice to ensure finite responses due to network effects. It is well known that a row normalization or network weights means to destroy the notion of absolute distance or neighborliness in the network. In other words, firms which exchange a large absolute amount of input and output with other firms are treated similarly to ones which exchange a small amount as long as the relative magnitude of the amount is similar. This is the consequence of row normalization as a vector normalization. Note that scalar normalizations of network matrices would avoid destroying the notion of absolute distance or neighborliness (see LeSage and Pace 2008; Kelejian and Prucha 2010, for further details on the normalization of network matrices).

5 Note that with firm-specific parameters on two tariff determinants, one lagged-own-dependent-variable parameter and one lagged-network-dependent-variable parameter apart from a firm-specific constant, the overall residual degrees of freedom of the proposed model will be **660,514** or about **187** for the average firm when using the data which will be described and used below. As only five parameters have to be estimated per firm there are many-enough residual degrees of freedom to principally estimate the parameters of interest at sufficiently high precision when pooling the data across events. Note that, irrespective of whether parameters are common to all firms or not, researchers who are interested in the standard error of these parameters would have to consider that the dependent variable of this second setup is estimated. Hence, for inference, one would have to pursue a weighted least-squares approach (see Saxonhouse 1977). The latter is typically ignored in work on the determinants of abnormal returns. Note that parameters such as $(\lambda ,\kappa ,\beta _1,\beta _2)$ are typically assumed to be common across firms in the finance literature (see Moser and Rose 2014). However, recent work in econometrics points to the potential importance of parameter heterogeneity across cross-sectional units when estimating panel-data models (see Christodoulou and Sarafidis 2017; Sarafidis and Wansbeek 2020). While it turned out feasible to estimate all parameters specific to the level of the firm, optimizing the degree of pooling along the lines of Christodoulou and Sarafidis (2017) turned out computationally infeasible within reasonable time due to the size of the cross section of firms at hand.

6 Recall that the estimation window where policy-shock effects on abnormal returns are estimated is typically short in order to ensure that no other influences exert a collinear impact on outcome (see Moser and Rose 2014).

7 We use more than just the USA–China exchange rate to reflect the multilateral trade exposure of firms in the country.

8 We also estimated a second-order model. We suppress the associated results here for brevity. *F* tests suggest that the data support the use of richer adjustment processes as captured by the model including a first, second, and third lag of abnormal returns over the first-order and also the second-order lag model. However, as the considered event windows are relatively short, we cannot estimate even richer models.
